# Adaptive Changes in Detoxification Metabolism and Transmembrane Transport of *Bombyx mori* Malpighian Tubules to Artificial Diet

**DOI:** 10.3390/ijms24129949

**Published:** 2023-06-09

**Authors:** Lijing Liu, Dongchao Zhao, Genhong Wang, Qingxiu He, Yuwei Song, Yulu Jiang, Qingyou Xia, Ping Zhao

**Affiliations:** 1 Biological Science Research Center, Integrative Science Center of Germplasm Creation in Western China (CHONGQING) Science City, Southwest University, Chongqing 400715, China; 2 Key Laboratory for Germplasm Creation in Upper Reaches of the Yangtze River, Ministry of Agriculture and Rural Affairs, Chongqing 400715, China

**Keywords:** transcriptome, metabolome, Malpighian tubules, artificial diet, silkworm (*Bombyx mori* L.)

## Abstract

The high adaptability of insects to food sources has contributed to their ranking among the most abundant and diverse species on Earth. However, the molecular mechanisms underlying the rapid adaptation of insects to different foods remain unclear. We explored the changes in gene expression and metabolic composition of the Malpighian tubules as an important metabolic excretion and detoxification organ in silkworms (*Bombyx mori*) fed mulberry leaf and artificial diets. A total of 2436 differentially expressed genes (DEGs) and 245 differential metabolites were identified between groups, with the majority of DEGs associated with metabolic detoxification, transmembrane transport, and mitochondrial function. Detoxification enzymes, such as cytochrome P450 (CYP), glutathione-S-transferase (GST), and UDP-glycosyltransferase, and ABC and SLC transporters of endogenous and exogenous solutes were more abundant in the artificial diet group. Enzyme activity assays confirmed increased CYP and GST activity in the Malpighian tubules of the artificial diet-fed group. Metabolome analysis showed increased contents of secondary metabolites, terpenoids, flavonoids, alkaloids, organic acids, lipids, and food additives in the artificial diet group. Our findings highlight the important role of the Malpighian tubules in adaptation to different foods and provide guidance for further optimization of artificial diets to improve silkworm breeding.

## 1. Introduction

The adaptability of insects to changing food sources is related not only to the physical properties of the food, such as smell and taste, but also to its nutritional and toxic components [1]. In addition to basic ingredients, such as mulberry leaf powder, inorganic salts, and vitamins, artificial diets also contain defatted soybean powder as an important source of protein, corn powder as an energy source, and other food additives such as propyl gallate [2,3]. However, studies have shown that soybean powder contains a variety of anti-nutritional factors that inhibit growth and development, including soybean saponins [4], isoflavones [5], tannins [6], soybean oligosaccharides [7], and antigen proteins [8]. Soybeans are also rich in triterpenoids and sesquiterpenoids. Ingestive exposure to xenobiotics, either natural or synthetic, present in food tends to induce strong metabolic resistance in insects [9]. In general, insects can adapt to food by directly avoiding contact through vision and smell [10] and may also adapt to food by active excretion and sequestration [9,11]. However, insects commonly adapt to food using detoxifying enzymes to metabolize the xenobiotics present in the food [12,13]. Metabolic detoxification of xenobiotics occurs in three phases [14,15]. The reaction in phase I is mainly performed by cytochrome P450 (CYP) and carboxylesterase, which catalyze the redox reactions and hydrolysis. After entering phase II, glutathione-S-transferase (GST) and UDP-glycosyltransferase (UGT) catalyze further conjugation reactions to generate less toxic or non-toxic metabolites for excretion or enter phase III [16,17], in which ABC transporters and cell membrane transporters secrete metabolites [18]. For example, volatile substances produced by plants, such as jasmonic acid and salicylic acid, induce cotton bollworms to produce excess CYP450, which can metabolize these substances prior to ingestion, representing an adaptative strategy to consume plants with such toxins [19]. Similarly, the green peach aphid, *Myzus persicae*, overproduces GST to facilitate their excessive consumption of glucosinolates and isothiocyanate-rich cruciferous plants [20].

With economic development, rapid adjustment of the industrial structure, and a shortage of the rural labor force, traditional silkworm (*Bombyx mori*) rearing with mulberry leaves (as their natural food source) is facing challenges. As a new rearing method, feeding them artificial diets has attracted substantial attention, which overcomes the limitations of the seasonal production of and pathogenic microorganisms on mulberry leaves, thereby enabling large-scale automated farming practices [2,21,22]. However, silkworms have acquired natural adaptations to mulberry leaves in the course of their co-evolution over thousands of years [23,24]. This natural habit of exclusively feeding on mulberry leaves has resulted in a seasonal pattern for silkworm rearing, which poses a challenge for the development of an appropriate artificial diet. Inappropriate food changes cause maladaptations in silkworms, manifesting as growth retardation, decreased immune resistance, and decreased silk quality [25,26]. Therefore, it is particularly important to explore the mechanisms underlying the metabolic adaptation of silkworms to artificial diets.

The Malpighian tubule is an important metabolic detoxification and transport organ in silkworms [27,28]. In addition to its classical osmoregulatory role, microarray analysis has shown that high levels of CYP, GST, and alcohol dehydrogenase, and numerous organic solute transporters, dominate the tubule transcriptome, which endows the Malpighian tubule with strong transport and excretion capabilities, thereby expanding its role in the metabolism and detoxification of endogenous solutes and xenobiotics [29,30,31]. *Helicoverpa zea* metabolically adapted to an artificial diet supplemented with capsaicin via mobilization of the detoxification enzyme UGT by the Malpighian tubules [32]. In addition, after honeybees consumed artificial diets containing the common alkaloid nicotine, the expression of the detoxification enzyme CYP was upregulated in phase I, whereas expression of the ABC transporter related to toxin clearance was upregulated in phase III [33]. However, the role of Malpighian tubules in the adaptation of silkworms to artificial diets, along with the specific biomolecules involved, remains unclear. Therefore, in this study, we aimed to systematically investigate this mechanism using transcriptome and metabolome analyses of the Malpighian tubules of silkworms after feeding with mulberry leaves or an artificial diet. The identification of key differentially expressed genes (DEGs) and metabolic components under artificial diet feeding provides new insights into the molecular mechanisms of insect adaptation to different foods and lays a theoretical foundation for further optimization of artificial diets.

## 2. Results

### 2.1. Effect of Diet on Silkworm Growth and Development Performance

**Figure 1:**
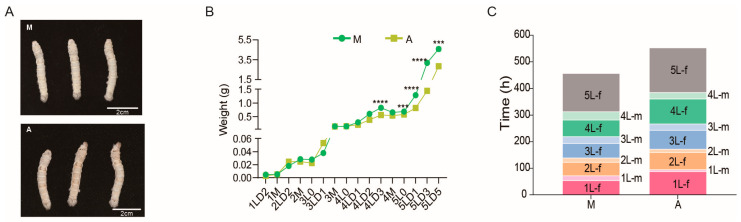
Effect of different diets on silkworm growth and development performance. (**A**) Silkworms at 0 h of the fifth-instar larval stage from the mulberry leaf-fed (M) and artificial diet-fed (A) groups. (**B**) Body weight of silkworms fed different diets measured from the second day of the first-instar stage to the fifth day of the fifth-instar stage (*n* = 30). (**C**) Development time of silkworms of different groups (*n* = 30). 1L, 2L, 3L, 4L, and 5L represent the first, second, third, fourth, and fifth instar, respectively. f, feeding; m, molting. *** *p* < 0.001, **** *p* < 0.0001 (Student’s *t*-test).

After fourth-instar molting, the body color of group M silkworms fed with mulberry leaf was white, whereas that of group A silkworms fed with artificial diet was yellow, indicating an unhealthy state (Figure 1A). There was no significant difference in body weight between the M and A groups from the first- to third-instar stage. However, from the end of the fourth instar to the fifth instar, the body weight of group A was markedly reduced compared with that of group M, especially on the first, third, and fifth days of the fifth-instar stage by 37%, 54%, and 38%, respectively (Figure 1B). In terms of growth and development parameters, the feeding time of group A silkworms was 119 h longer, the molting time was 23 h shorter, and the full-age development duration was approximately 98.5 h slower than that of the M group silkworms (Figure 1C).

### 2.2. Malpighian Tubules Show Distinct Transcriptomic Alterations in Response to Diet Change

**Figure 2:**
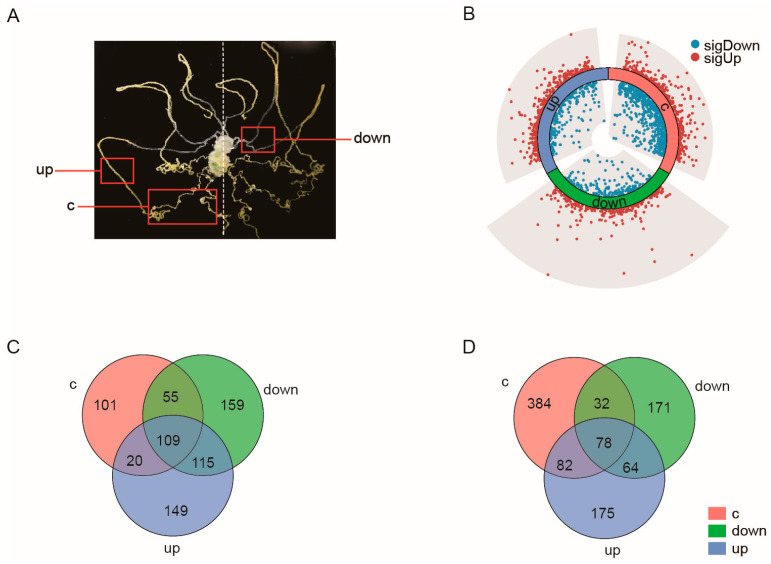
Morphology of different anatomical regions of the Malpighian tubule and transcriptomic analyses from the mulberry leaf-fed (M) and artificial diet-fed (A) groups. (**A**) The Malpighian tubule is subdivided into three regions according to morphological, color, and functional differences: upward curly section (c), upward straight section (up), and downward section (down). (**B**) Volcano plots of differentially expressed genes (DEGs) identified between the A and M groups. (**C**) Venn diagram of upregulated DEGs in the three Malpighian tubule regions. (**D**) Venn diagram of downregulated DEGs in the three Malpighian tubule regions.

Sequence information and mapping results for the transcriptome of the three sections of the Malpighian tubules (Figure 2A) are presented in Table S1 and Figure S1. We identified 861, 837, and 738 DEGs in the upward curly, upward straight, and downward regions, respectively (Table S2). The volcano map in Figure 2B shows the overall distribution of DEGs. The Venn diagram shows that 15.4% and 7.9% of the upregulated and downregulated DEGs, respectively, were common to the three regions; thus, the majority of DEGs were region-specific (Figure 2C,D).

**Figure 3:**
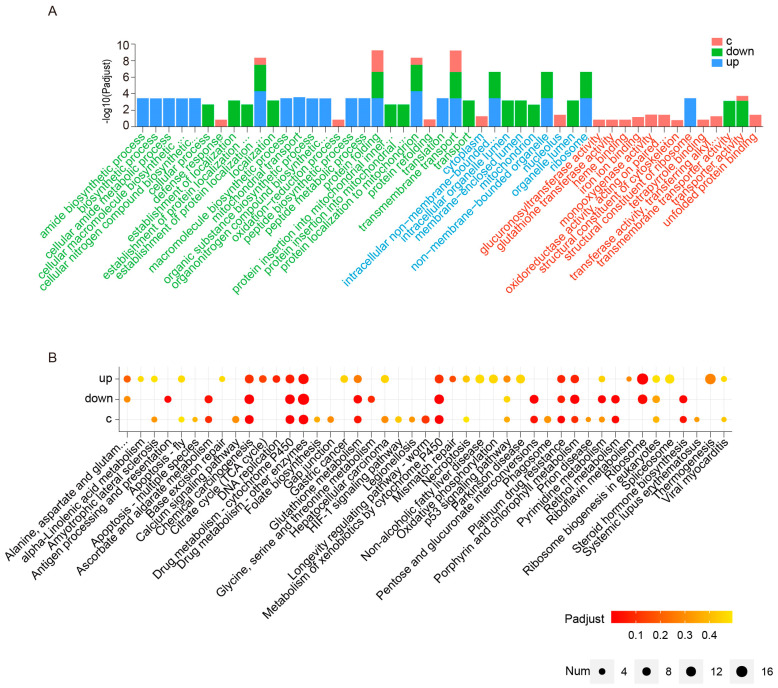
Gene Ontology (GO) and Kyoto Encyclopedia of Genes and Genomes (KEGG) functional enrichment analysis for differentially expressed genes (DEGs) in the three regions of the Malpighian tubule (c = upward curly section, up = upward straight section, and down = downward section). (**A**) Histogram of GO classification terms of upregulated DEGs. Only the top 15 terms enriched by the GO analysis are displayed for each region. The horizontal axis represents the GO category, and the vertical axis represents the enrichment score [−log (*p* adjusted)] of the term. Green, blue, and red text represent biological processes, cellular components, and molecular functions, respectively. (**B**) KEGG enrichment analysis scatterplot. The color of the circle indicates the adjusted *p*-value, and the size of the circle indicates the number of DEGs in the functional pathway. Pathways with an adjusted *p*-value < 0.05 were considered significantly enriched.

GO enrichment analysis showed that the DEGs were primarily associated with transport, ribosomes, and mitochondria. Transmembrane transport was the most significantly enriched biological process, followed by protein folding, establishment of protein localization to the mitochondria, and protein localization to the mitochondria. Molecular function analysis showed that these genes are involved in transporter activity, ribosome structural constituents, and transmembrane transporter activity. Cell components analysis indicated that the DEGs in the Malpighian tubules after artificial diet feeding were closely associated with the ribosomes (non-membrane-bound organelles and intracellular non-membrane-bound organelles) (Figure 3A).

Furthermore, KEGG pathway enrichment analysis identified multiple detoxification-related metabolic pathways, including drug metabolism-other enzymes, drug metabolism-cytochrome P450, metabolism of xenobiotics by cytochrome P450, chemical carcinogenesis, glutathione metabolism, and platinum drug resistance, in all three regions of the Malpighian tubules. In addition, porphyrin and chlorophyll metabolism and ribosome biogenesis in eukaryotes were upregulated KEGG pathways in all three regions. The ribosomal pathway was only significantly enriched in the upward straight and downward regions (Figure 3B).

### 2.3. Analysis of the Main DEGs

**Figure 4:**
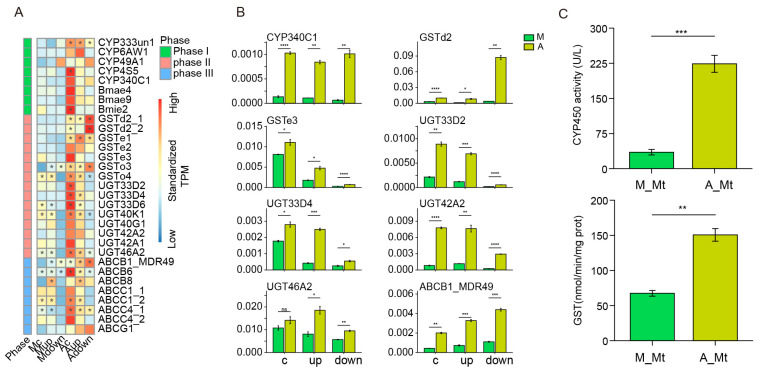
Analysis of differentially expressed genes (DEGs) from the detoxification pathway and determination of related enzyme activities. (**A**) Heatmap of DEGs related to the detoxification process. The green, red, and blue lines represent phase I, phase II, and phase III of detoxification, respectively. The expression data are normalized along the rows; the redder the box, the higher the expression level. The asterisk indicates that the expression level (TPM value) is greater than 20. (**B**) Quantitative polymerase chain reaction validation of eight randomly selected genes. (**C**) Determination of CYP and GST enzyme activities in the Malpighian tubules. Error bars indicate ± standard error of the mean. * *p* < 0.05, ** *p* < 0.01, *** *p* < 0.001, **** *p* < 0.0001, ns, no significant difference (Student’s *t*-test). A, artificial diet-fed group; M, mulberry leaf-fed group; Mt, Malpighian tubules; c upward curly section; up, upward straight section; down, downward section.

The heatmap in Figure 4A shows that the expression levels of several genes related to detoxification metabolism in silkworms fed artificial diets were higher than those in silkworms fed mulberry leaves. The detoxification system in the silkworm involves five CYP genes [34] and three carboxylesterase [35] genes in phase I; seven GST and eight UGT [36,37] genes in phase II; and eight ABC genes in phase III [38]. Among them, the TPM values of GSTd2_1, GSTo4, UGT33D6, UGT40K1, and ABCB1_MDR49, in some regions, were higher than 100 in group A silkworms, and these transcripts were upregulated by approximately 14-, 2-, 5-, 2-, and 3-fold, respectively, compared with the corresponding levels in the group M silkworms. Moreover, the expression levels of most genes were higher in the upward curly region than those in the upward straight and downward regions of the Malpighian tubules.

To further validate the accuracy of the RNA-sequencing data, we randomly selected eight genes for RT-qPCR analysis, and the results were consistent with the RNA-sequencing data (Figure 4B). Furthermore, enzyme activity assays showed that the activities of CYP and GST in the Malpighian tubules of silkworms fed artificial diets were significantly higher than those in silkworms fed mulberry leaves (Figure 4C). These enzyme activities were also higher in the fat body and midgut of the silkworms in group A, which are important detoxification organs (Figure S2).

**Figure 5:**
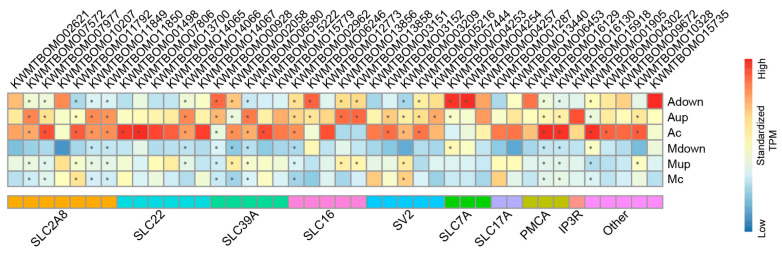
Expression of transmembrane transport-related genes. The expression data are normalized along the rows; the redder the box, the higher the expression. The asterisk indicates that the expression level (TPM value) is greater than 20.

In addition to ABC transporters, organic solute transporters have been shown to dominate the Malpighian tubule transcriptome and endow the Malpighian tubules with the broadest capacity for the excretion of organic solutes and xenobiotics [30]. Given that we identified transmembrane transport as one of the most significantly enriched GO terms, we further focused on the DEGs related to transmembrane transport. A large number of solute carrier (SLC) family members were among the upregulated transmembrane transporters, the most abundant of which was *SLC2A8* [39], also known as *GLUT8*, which belongs to the family of facilitative trehalose transporters with a total of seven members. Other upregulated SLC genes after artificial diet feeding included *SLC22* (encoding organic cation/anion/zwitterion transporters) [40], *SLC39A* (Zrt/Irt-like protein family of zinc transporters) [41], and SLC16 (monocarboxylate transporters). Moreover, five synaptic vesicle glycoprotein 2 genes [42], three SLC7A genes, two SLC17A genes, and five other transporters were upregulated in the Malpighian tubules of group A silkworms. Furthermore, the expression of plasma membrane calcium-transporting ATPase (*PMCA*) and inositol 1,4,5-trisphosphate receptor (*IP3R*), which are involved in the transport of calcium ions, was upregulated in group A silkworms, suggesting that calcium ions may be involved in the regulation of Malpighian tubule function under artificial feeding conditions (Figure 5).

**Figure 6:**
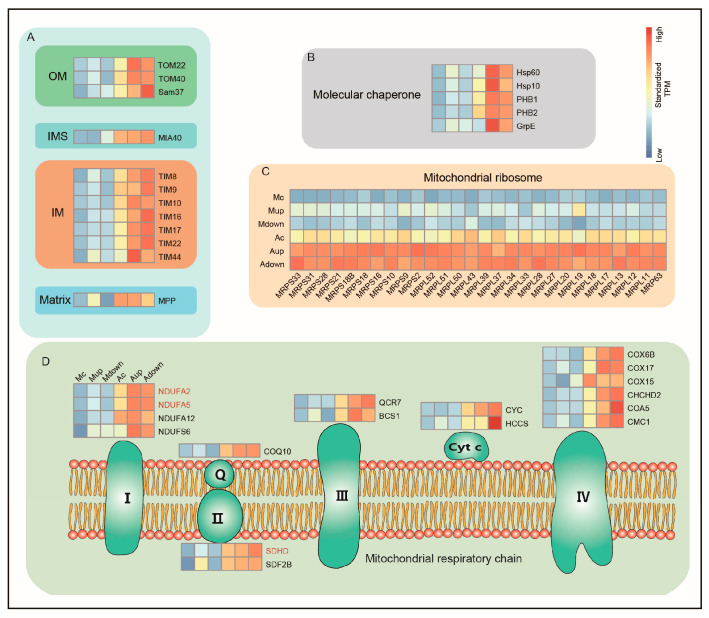
Gene expression profile of mitochondria-related genes. (**A**) Genes involved in nuclear-encoded mitochondrial precursor protein import. (**B**) Mitochondrial molecular chaperone-associated differentially expressed genes. (**C**) Expression of mitochondrial ribosomal protein-related genes. (**D**) Diagram of the respiratory chain and expression of genes related to the respiratory chain complex. The color scale from blue to red in the heatmap indicates the expression level (TPM value) from low to high. Respiratory enzymes encoded by the mitochondrial genome are indicated in red. Complex I: nicotinamide adenine dinucleotide ubiquinone reductase (NADH dehydrogenase); Complex II: succinate ubiquinone oxidoreductase; Complex III: ubiquinone cytochrome oxidoreductase; Complex IV: cytochrome c oxidase; Q: ubiquinone; Cyt c: cytochrome c. PHB1, prohibitin protein WPH; PHB2, mitochondrial prohibitin complex protein; GrpE, grpE protein homolog; NDUFA2, 5, 12 NADH dehydrogenase [ubiquinone] 1 alpha subcomplex subunit 2, 5, 12; NDUFS6, NADH dehydrogenase [ubiquinone] iron-sulfur protein 6; COQ10, coenzyme Q-binding protein COQ10 homolog A; SDHD, succinate dehydrogenase [ubiquinone] cytochrome b small subunit; SDF2B, succinate dehydrogenase assembly factor 2-B; CYC, cytochrome c; HCCS, cytochrome c-type heme lyase; COX6B, cytochrome c oxidase, subunit VIb; COX17, cytochrome c oxidase copper chaperone; COX15, cytochrome c oxidase assembly protein; CHCHD2, coiled-coil-helix-coiled-coil-helix domain-containing protein 2; COA5, cytochrome c oxidase assembly factor 5; CMC1, cytochrome c oxidase biogenesis protein.

Enrichment analysis also showed that the biological process of protein localization to the mitochondria was more active in the Malpighian tubules of group A silkworms than that in group M silkworms. Therefore, we further examined the expression profile of mitochondrial-related genes. Multiple genes related to the import of nuclear-encoded mitochondrial precursor proteins into the mitochondria were upregulated after artificial diet feeding, including the genes encoding receptors of the translocase of the outer mitochondrial (TOM) membrane complex (TOM22 and TOM40), mitochondrial outer membrane transport complex SAM37, mitochondrial intermembrane space assembly machinery (MIA40), seven mitochondrial import inner membrane translocases (TIM) genes, and a mitochondrial processing peptidase (MPP). After the precursor protein enters the mitochondria, it must be folded correctly to function, which depends on molecular chaperones [43]. In line with this, we found that the expression of the molecular chaperones *HSP10* and *HSP60* were among the upregulated genes in group A silkworms, along with the molecular chaperones *PHB1*, *PHB2*, and *GrpE*. Remarkably, genes encoding mitochondrial ribosomal proteins (MRPs) belonging to the structural and non-catalytic components of the ribosome [44] were also upregulated, including 10 small MRP subunits (MRPSs), 17 large MRP subunits (MRPLs), and 1 MRP63 subunit, all of which are encoded by the nuclear genome and then transported to the mitochondria [45]. The aforementioned translocase complexes may be involved in this process. Furthermore, these upregulated ribosomal proteins were highly expressed in the upward straight and downward regions. As the functional core of mitochondria, the respiratory chain (electron transport chain) is located in the inner mitochondrial membrane and is composed of a series of electronic carriers [46]. Analysis of the main members of the respiratory chain showed that genes encoding four complex I, two complex II, one coenzyme Q, two complex III, two cytochrome c, and six complex IV proteins were upregulated in group A silkworms (Figure 6).

### 2.4. Differentially Abundant Metabolites and Comparative Analysis of Transcription and Metabolism in the Malpighian Tubules in Response to Artificial Diet Feeding

**Figure 7:**
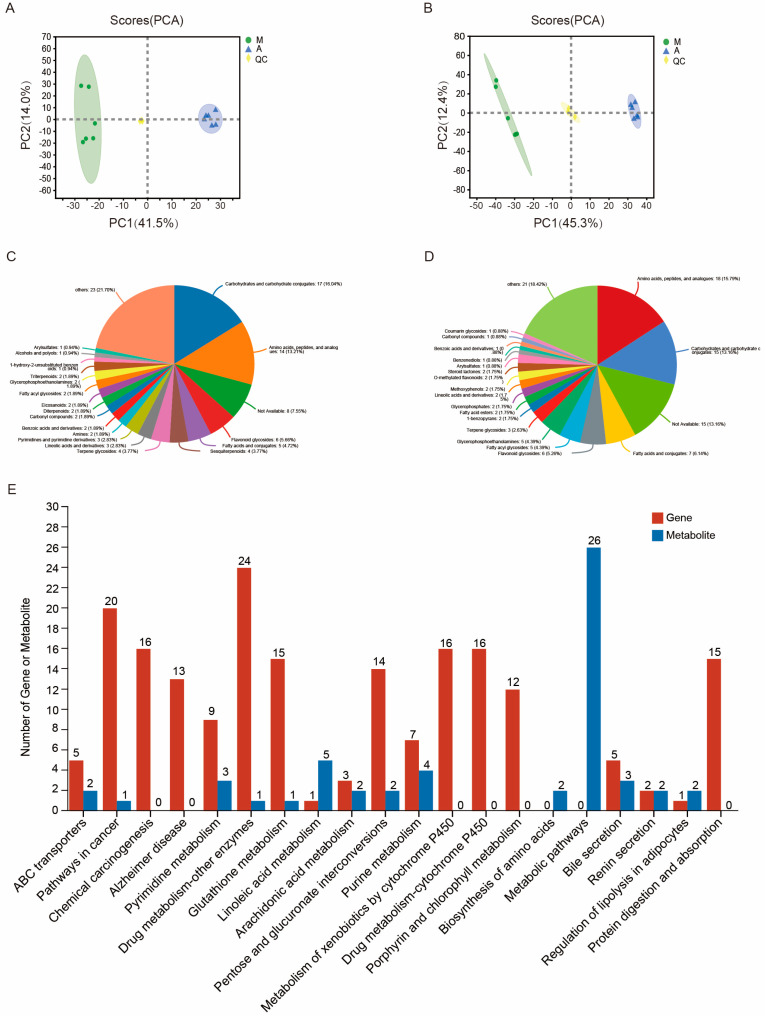
Metabolomics and comparative transcriptomic and metabolomic analysis of the artificial diet-fed (A) versus mulberry leaf-fed (M) groups. (**A**, **B**) Principal component analysis (PCA) score plot of metabolites determined in (A) positive-ion mode and (B) negative-ion mode. Different colors represent samples from silkworms fed different diets. (**C**) Pie chart showing the categories and relative proportions of upregulated (**D**) and downregulated metabolites in the Malpighian tubules of silkworms raised on the artificial diet. (**E**) Histogram of Kyoto Encyclopedia of Genes and Genomes (KEGG) pathway enrichment of differential genes/metabolites. The horizontal coordinates represent the KEGG pathway name, and the vertical coordinates represent the number of genes/metabolites.

LC-MS/MS identified 382 and 678 metabolites in positive- and negative-ion mode, with a total of 83 and 162 differential metabolites, respectively, between the 2 groups (Table S3). Cluster heatmap analysis classified these metabolites into 10 groups (Figure S3). PCA showed that the metabolites of group A were clearly separated from those of group M, indicating that Malpighian tubule metabolism was significantly altered by the artificial diet (Figure 7A,B). The HMDB classification of differential metabolites showed that “carbohydrates and carbohydrate conjugates” and “amino acids, peptides, and analogs” were the most abundant categories in the up- and down- regulated metabolites, accounting for 29.25% and 28.95% of all differential metabolites, respectively (Figure 7C,D). In addition, terpenoids, such as sesquiterpenoids, triterpenoids, and diterpenoids, were abundant among the upregulated metabolites, and lipids among the downregulated metabolites included fatty acids and conjugates, glycerophosphoethanolamines, steroid lactones, lineolic acids and derivatives, and fatty acid esters. The significantly upregulated metabolites based on VIP > 1.5 and FC > 2 were further screened. In addition to six terpenoids, the contents of one benzoic acid and its derivatives, two carbonyl compounds, two organic acids and their derivatives, seven lipids, four flavonoids, and two alkaloids increased significantly in the Malpighian tubules of silkworms raised on artificial diets (Table 1). Among them, N-propyl gallate, a common food antioxidant, showed the highest increase (564-fold), followed by 3-hydroxy-2-(4-methylbenzoyl)-4H-1-benzopyran-4-one (110-fold) and triterpenoid 3-beta-3-hydroxy-18-lupen-21-one (104-fold).

To further evaluate the effects of transcriptome changes on the metabolome, differentially expressed genes and different accumulated compounds were subjected to KEGG pathway analysis, and the top 10 KEGG pathways with the largest number of differential genes and metabolites were compared. Of these, the metabolic pathways, drug metabolism-other enzymes, cancer pathways, glutathione metabolism, chemical carcinogenesis, pentose and glucuronate interconversions, metabolism of xenobiotics by cytochrome P450, and drug metabolism-cytochrome P450 were significantly enriched (Figure 7E).

## 3. Discussion

### 3.1. Activation of a Fully Mobilized Detoxifying Enzyme System for Metabolic Adaptation to an Artificial Diet

Insects have evolved intricate metabolic adaptations to tolerate potential toxins in plants, enabling broader dietary options to facilitate diversity and inhabiting various environments. Luque et al. [17] reported that silkworm UGT has broad substrate specificity for flavonoids, coumarins, terpenoids, and simple phenols. The induction of CYP and GST by allelochemicals, such as terpenoids, flavonoids, and alkaloids, has also been reported [47,48,49]. Here, we identified that various detoxification genes related to all three phases of metabolic detoxification were significantly upregulated in the Malpighian tubules of group A silkworms. Therefore, these genes may be used to metabolize different kinds of harmful or useless substances present in the artificial diet. Based on the abundance of upregulated genes and enzyme activity results, CYP, GST, and UGT may play particularly important roles in this process. In Lepidoptera, members of the ABC transporter B, C, and G subfamilies are mainly involved in xenobiotic resistance [18]. The expression levels of detoxification-related genes (such as *Cyp12d1* and *GstE1*) and transport-related genes (such as *MET* and *dMRP*) were reported to increase in the Malpighian tubules of *Drosophila melanogaster* in response to dietary exposure to toxins [50]. Our transcriptome analysis showed that multiple ABC transporters in these subfamilies, such as MDR49 [18,38], ABCC1, and ABCC4 [51], were upregulated in the Malpighian tubules after silkworms were fed an artificial diet. However, the exact correspondence between detoxification enzymes and allelochemicals has not been clarified yet and requires further in-depth research.

The main ingredients added to the artificial diet of silkworms were soybean meal, corn flour, and mulberry leaf powder [26]. Beans are rich in several types of secondary metabolites, such as flavonoids and isoflavones among phenolics and terpenoids, especially triterpenoids, steroidal saponins, and tetraterpenes [52,53]. Corn, as a gramineous plant, is also rich in secondary metabolites, such as phenylpropanoids and alkaloids [54]. Some of these dietary components, such as the triterpenoid 3-beta-3-hydroxy-18-lupen-21-one and the flavonoid cycloalliin, were significantly upregulated in the Malpighian tubules of group A silkworms, indicating that they may be directly derived from the soybean meal and corn flour in the diet. This implies that some useless materials present in the food or food metabolites are directly excreted by the Malpighian tubules in the silkworm *B. mori*. Propyl gallate is an antioxidant widely used in foods, pharmaceuticals, and cosmetics [55]. Propyl gallate is also commonly added to an artificial diet to protect oils and fat-containing foods from the rancidity caused by peroxide formation [56]. We also found a large amount of propyl gallate among the metabolites of the Malpighian tubules in silkworms reared on artificial diets. A previous study also showed the presence of propyl gallate in the metabolites of the intestine, hemolymph, and silk glands of silkworms fed artificial diets, which had a negative effect on silk production [57]. These results strongly suggest that propyl gallate is harmful to silkworms. Therefore, the selection of antioxidants with better tolerance in silkworms is a future direction for the optimization of artificial diets.

Ribosome biogenesis, especially 90S pre-ribosome and rRNA modification-related genes, plays an extremely important role in the silkworm detoxification process [58]. We found that several genes belonging to the 90S pre-ribosome complex and those associated with rRNA modifications, such as *NOP1*, *SNU13*, and *NHP2*, were upregulated after artificial diet feeding (Table S4), implying that these genes also play a role in the metabolism and detoxification processes performed by the Malpighian tubules in silkworms reared with artificial diets.

### 3.2. Artificial Diets Induce Transmembrane Transport Function in the Malpighian Tubules of Silkworms

The SLC family is the second largest family of membrane proteins which mediates the flow of various substances, such as sugars, amino acids, nucleotides, inorganic ions, and drugs, across cell membranes [59]. Furthermore, SLC22 family genes function in the renal excretion of drugs, xenobiotics, and endogenous compounds [40]. GLUT8 belongs to the SLC2A family and is involved in high-affinity glucose transport in *Xenopus* oocytes [60]. GLUT8 is also localized to the podocytes and distal tubular epithelial cells of the mouse kidney, suggesting a role as a regulator of glucose homeostasis in different cellular compartments within the mouse cell [61]. We found several genes of the GLUT8 and SLC22 families with upregulated expression in the Malpighian tubules of silkworms reared on artificial diets, indicating a requirement for more urgent or greater material transport under these conditions. The SLC39A/ZIP transporter is involved in the regulation of intracellular zinc homeostasis and is responsible for the transport of zinc from the extracellular space or organelles to the cytoplasm [62,63]. The upregulated expression of several ZIP transporters, including ZIP10 (KWMTBOMO00928) and ZIP11 (KWMTBOMO06580), under the artificial diet may suggest a need to increase the cytosolic concentration of zinc ions in the Malpighian tubules to maintain physiological function. Overall, the upregulation of a large number of transporters implies that substance metabolism in the Malpighian tubules of silkworms is severely altered by artificial diet feeding and, therefore, needs to be rebalanced.

### 3.3. Artificial Diets Activate Mitochondrial Function in the Malpighian Tubules of Silkworms

The main function of the mitochondria is to provide energy for various biological processes through oxidative phosphorylation, which is mainly regulated by the mitochondrial respiratory chain composed of complexes I–V [64]. We found that several members of the respiratory chain involved in oxidative phosphorylation were consistently upregulated in the Malpighian tubules of group A silkworms, implying a higher energy requirement. Among the members of the respiratory chain, CHCHD2 is a regulator of mitochondrial function that can bind and regulate the activity of cytochrome C oxidase (COX) and regulate the expression of the COX4I2 subunit in the nucleus [65,66]. The upregulation of *CHCHD2* expression in the Malpighian tubules of group A silkworms also indicates higher cellular energy requirements after artificial diet feeding. The mitochondrial ribosome is a large ribonucleoprotein complex responsible for protein synthesis in the mitochondria, which is structurally supported by MRPs [44,67]. We identified that several MRPs, including 10 MRPSs and 17 MRPLs, were upregulated in the Malpighian tubules of group A silkworms, indicating a higher rate of mitochondrial translation and increased mitochondrial biogenesis. MRPs and members of the respiratory chain are only partially synthesized in the mitochondria, with most synthesized as precursors in the cytoplasm and then imported through the mitochondrial import complex (TOM and TIM) [45,68]. The upregulation of multiple TOMs and TIMs in the Malpighian tubules of the artificial diet group implies that more mitochondria-associated proteins in this group undergo TOM- and TIM-dependent mitochondrial import, thereby indicating more active mitochondrial function. This could be explained by the high amount of energy required in the transport of various substrates through the bilayer of biological lipids, including for the excretion of toxic substances in the metabolic detoxification process by ABC transporters or for the transmembrane transport of high amounts of organic solutes and xenobiotics by SLC transport family proteins [69], thus requiring more active function of mitochondria in the Malpighian tubules to process the components of artificial diets.

## 4. Materials and Methods

### 4.1. Insects and Diets

Larvae of the hybrid silkworm strain Liangguang II used in this experiment were provided by Shandong Guangtong Silkworm Seed Co., Ltd. (Qingzhou, China). The mulberry leaves were harvested from an experimental field at Southwest University (Chongqing, China). The artificial diet was developed in our laboratory (Biological Science Research Center, Southwest University, Chongqing, China). The raw materials included 30% mulberry leaf powder, 35% defatted soybean meal, 20% corn flour, 5% forming agent, 5% vitamins and inorganic salt complexes, and 5% other components [3]. The diets were prepared with sterile water at a ratio 2.1 times the dry weight of the diet and heated at 100 °C for 50 min. The processed feed was then cooled naturally and stored at 4 °C for future use within one week.

### 4.2. Insect Rearing

Silkworms were randomly divided into two groups that were either fed fresh mulberry leaves (group M) or the artificial diet (group A) at the instar stage. The silkworms in group M were fed three times a day to ensure a sufficient supply of fresh mulberry leaves. The silkworms in group A were fed once from the newly hatched period to the second-instar stage, and were then fed two, two, and three times a day at the third-, fourth-, and fifth-instar stage, respectively. All silkworms were reared under the same temperature conditions: 27 ± 1 °C for the first to third instars and 25 ± 1 °C for the fourth to fifth instars. The relative humidity was maintained at 70% ± 5% for the M group and at 80% ± 5% for the A group.

### 4.3. Developmental Parameters Measurement and Sample Collection

The developmental time and body weight of all instars were investigated. The fresh weight of the larvae (*n* = 30) was measured at the same developmental stage from 48 h after hatching to day 5 of the fifth-instar stage. The silkworm larvae were fixed and cut in a dissecting pan with surgical nippers and scissors. The Malpighian tubules were collected on ice from the larvae on day 3 of the fifth instar, as this is the most active developmental stage for the silkworm [57]. The samples were rapidly frozen in liquid nitrogen and stored at –80 °C for subsequent transcriptomic and metabolomic profiling analyses.

### 4.4. RNA Preparation and Transcriptome Sequencing

Total RNA was extracted from the Malpighian tubules of fifth-instar larvae of the M and A groups using TRIzol reagent (Invitrogen, Carlsbad, CA, USA) The Malpighian tubules of each group were divided into three sections: upward curly, upward straight, and downward according to the morphology and direction of excreta movement, with three biological replicates for each section represented by more than 20 larvae per biological replicate. RNA-sequencing was performed using an Illumina NovaSeq 6000 platform by Shanghai Majorbio Bio-pharm Biotechnology Co., Ltd. (Shanghai, China). The original sequencing data generated in this study have been deposited in the National Center for Biotechnology Information Short Read Archive (accession number: PRJNA923857).

### 4.5. Transcriptome Data Analysis

The raw sequencing reads were filtered to obtain clean reads by removing adapters, poly-N–containing reads, and low-quality reads. The Phred scores (Q20 and Q30) and GC content of the clean reads were calculated, and all subsequent analyses were based on high-quality clean data. Reference genome and gene model annotation files were downloaded directly from the Silkworm Genome Database (https://kaikobase.dna.affrc.go.jp/KAIKObase_download.html, accessed on 2 June 2023). Gene expression levels were quantified as transcript-per-million (TPM) using the RSEM package (version 1.2.23) [70]. Significant differentially expressed genes (DEGs) between the M and A groups were identified using DESeq2 [71] according to an adjusted *p*-value ≤ 0.05 and |log_2_ (fold change)| ≥ 1. In addition, Gene Ontology (GO) enrichment and Kyoto Encyclopedia of Genes and Genomes (KEGG) pathway analysis of DEGs was performed using Goatools [72] and KOBAS software [73], respectively; GO terms and KEGG pathways with an adjusted *p*-value < 0.05 were considered to be significantly enriched. A bubble-and-bar diagram was used for visualization of the annotated terms and pathways.

### 4.6. Reverse Transcription-Quantitative Polymerase Chain Reaction (RT-qPCR)

Total RNA was extracted as described above, and 1 µg RNA was reverse transcribed to complementary DNA using the PrimeScript™ RT reagent Kit with gDNA Eraser (Takara, Japan). The qPCR analysis was then performed using SYBR qPCR Super MixPlus Kit (Novoprotein, Shanghai, China), according to the manufacturer’s instructions. Relative gene expression was normalized to that of the reference gene *eFL4A*, and each detection was performed at least three times. The primer sequences for the target genes are shown in Table S5.

### 4.7. Measurement of Metabolites

Malpighian tubule samples were accurately weighed to 100 mg, and the metabolites were extracted using a 400 µL of methanol:water (4:1, *v*/*v*) solution with 0.02 mg/mL L-2-chlorophenylalanin as the internal standard. The mixture was ground with a Wonbio-96c (Shanghai wanbo biotechnology Co., Ltd., Shanghai, China) high-throughput tissue crusher (50 Hz, 6 min) and all homogenates were extracted via ultrasonic extraction at 40 kHz for 30 min at 5 °C. Homogenates were incubated at –20 °C for 30 min to precipitate the proteins. After 30 min of centrifugation at 13,000× *g* at 4 °C, supernatants were carefully collected into sample vials for liquid chromatography-tandem mass spectrometry (LC-MS/MS) analysis. All samples were analyzed using the ultra-high-performance liquid chromatography triple time-of-flight system from AB SCIEX. All reagents were purchased from Invitrogen Corporation (Thermo Fisher Scientific, Carlsbad, CA, USA). The separation was performed on an HSS T3 column (100 mm × 2.1 mm, 1.8 μm). The mobile phase comprised 0.1% formic acid in water:acetonitrile (95:5, *v*/*v*) (solvent A) and 0.1% formic acid in acetonitrile:isopropanol:water (47.5:47.5, *v*/*v*) (solvent B). The column temperature was maintained at 40 °C and the injection volume was 10 µL. The gradient profile was as follows: 0–0.5 min (100% phase A), 0.5–2.5 min (0% to 25% phase B), 2.5–9 min (25% to 100% phase B), 9–13 min (100% phase B), 13–13.1 min (100% to 0% phase B), and 13.1– 16 min (100% phase A).

### 4.8. Metabolome Data Analysis

After mass spectrometry detection, the raw data were preprocessed using the Progenesis QI software version 2.4 (Waters Corporation, Milford, MA, USA; http://www.nonlinear.com/progenesis/q). The metabolites were searched and referenced to the Human Metabolome Database (HMDB; http://www.hmdb.ca/), METLIN (https://metlin.scripps.edu/), and Majorbio database. Multivariate statistical analyses, including principal component analysis (PCA) and orthogonal projections to latent structures discriminant analysis (OPLS-DA), were performed using the R package ropls version 1.6.2. In addition, Student’s *t*-tests and fold-difference analyses were performed. The metabolites significantly distinguishing the two diet groups were identified according to a variance in projection (VIP) value > 1 of the first principal component in the OPLS-DA model and *p* < 0.05 in the Student’s *t*-test. Pathway analysis was performed using the KEGG database (http://www.genome.jp/kegg/). Enrichment analysis was performed using Python 2.7 scipy.stats, and significantly enriched pathways were identified using Fisher’s exact test.

### 4.9. GST and CYP Activity Assays

GST enzyme activity was measured using a GST assay kit (Geruisi, Suzhou, China), following the manufacturer’s instructions. In brief, 10 μL of the extracts was added to 190 μL of the reaction mixture comprising reduced glutathione and 1-chloro-2,4-dinitrobenzene (CDNB). Enzymatic activity was measured at 340 nm over a 2-min interval at 25 °C. The protein concentrations of the extracts were quantified using a BCA Protein Assay Kit (Beyotime, Shanghai, China) with bovine serum albumin as the standard. One unit of GST activity was defined as the amount of enzyme required to conjugate 1 nmol of CDNB per minute per milligram of protein.

CYP450 activity was evaluated using an enzyme-linked immunoassay kit (Jiangsu Feiya Biotechnology Co., Ltd., Yancheng, China), according to the manufacturer’s protocol. Fresh samples were collected and homogenized in phosphate-buffered saline. The mixture was centrifuged at 3000× *g* for 10 min, and the supernatant was directly analyzed at 450 nm using a standard dilution curve. The extracts were collected for protein measurement. Three biological replicates were used for each assay.

## 5. Conclusions

In summary, transcriptome and metabolome analyses of silkworm Malpighian tubules revealed that silkworms adapt to food changes by regulating detoxification, transmembrane transport, and mitochondrial functions. The growth and development of the silkworm, *Bombyx mori*, was affected by artificial diet, especially at the fifth-instar stage. It may be due to the longest duration and the largest amount of food intake for the fifth instar, and the influence of artificial diet feeding on growth and development may have a cumulative effect. These results provide a reference for further in-depth analysis of the metabolic adaptation mechanism of insects to food, along with guidance for optimizing the formula of artificial diets for silkworms.

## Figures and Tables

**Table 1:** Significantly upregulated metabolites in the Malpighian tubules of silkworms raised on artificial diets.

Class	Metabolite	VIP	FC(A/M)	*p*-Value
Benzoic acids and derivatives	N-Propyl gallate	2.8115	564.9008	2.64 × 10^−14^
Carbonyl compounds	3-Hydroxy-2-(4-methylbenzoyl)-4H-1-benzopyran-4-one	2.0746	110.4676	6.74 × 10^−13^
	2′,3′,4′-Trihydroxyacetophenone	1.9507	3.5929	4.06 × 10^−8^
terpenoids	3beta-3-Hydroxy-18-lupen-21-one	2.7671	104.2965	1.17 × 10^−10^
	Shyobunyl acetate	2.4734	10.115	1.64 × 10^−11^
	Cincassiol B	2.2756	7.2251	7.75 × 10^−12^
	4,5-Dihydrovomifoliol	2.2691	4.1957	2.31 × 10^−7^
	Kessyl glycol	2.1987	2.9575	1.66 × 10^−11^
	Acorusnol	1.4963	2.0766	8.03 × 10^−5^
Organic acids and derivatives	N-[2-(3,4-dimethoxyphenyl)ethyl]-3-[4-methoxy-3-(sulfooxy)phenyl]prop-2-enimidic acid	2.4091	71.6817	4.78 × 10^−14^
	2-Methylcitric acid	2.4296	20.3789	7.10 × 10^−14^
lipids	7b-Hydroxy-3-oxo-5b-cholanoic acid	2.2763	31.4158	3.04 × 10^−8^
	Heptadecanoyl carnitine	2.2827	8.0294	1.45 × 10^−13^
	19-Norandrosterone	2.0716	7.7129	2.89 × 10^−10^
	13-Oxo-9,11-tridecadienoic acid	2.115	5.1548	3.97 × 10^−9^
	5,6-Dihydroxyprostaglandin F1a	2.1642	4.8017	4.49 × 10^−8^
	Hexadecanedioic acid	1.6981	3.3842	1.02 × 10^−5^
	Prostaglandin E2	1.9552	3.0415	1.33 × 10^−11^
Carbohydrates and carbohydrate conjugates	[6]-Gingerdiol 4′-*O*-beta-D-glucopyranoside	1.9581	14.7255	6.27 × 10^−12^
	Domesticoside	2.071	5.9443	3.48 × 10^−9^
	Lacosamide-glucuronide	1.5549	2.588	8.18 × 10^−7^
flavonoids	Cycloalliin	2.5788	7.4799	2.38 × 10^−16^
	Austalide K	1.9494	2.583	2.55 × 10^−10^
	Pelargonidin 3-*O*-glucoside	1.8203	2.3313	1.38 × 10^−7^
	Apigetrin	2.3895	2.0253	4.76 × 10^−13^
amino acids and their derivatives	Lysyl-Lysine	1.9195	7.6448	1.51 × 10^−8^
	Kinetensin 4-7	1.9371	4.1428	1.96 × 10^−12^
	Phenylalanyl-Arginine	1.8862	2.721	3.52 × 10^−9^
Alkaloids	3-Carboxy-1-hydroxypropylthiamine diphosphate	2.2551	4.7787	1.28 × 10^−9^
	1-(1,2,3,4,5-Pentahydroxypent-1-yl)-1,2,3,4-tetrahydro-beta-carboline-3-carboxylate	1.8015	4.5259	4.51 × 10^−9^
Terpene glycosides	Glucosyl (2E,6E,10x)-10,11-dihydroxy-2,6-farnesadienoate	1.8158	4.5214	2.52 × 10^−8^
	Tsangane L 3-glucoside	1.6478	2.5489	2.65 × 10^−5^

## Data Availability

Not applicable.

## Supplementary Materials

The following supporting information can be downloaded at: https://www.mdpi.com/article/10.3390/ijms24129949/s1.
