# Supplementary material for: Adaptive Changes in Detoxification Metabolism and Transmembrane Transport of Bombyx mori Malpighian Tubules to Artificial Diet
Source: Int J Mol Sci. 2023 Jun 9;24(12):9949. doi: 10.3390/ijms24129949 (PMC10298027; doi:10.3390/ijms24129949)
Supplement: Supplementary file 1 [file ijms-24-09949-s001.zip › Supplementary Material.pdf]

Supplementary Material

Figure S1. Histograms of six major databases (GO, KEGG, COG, NR, Swiss-Prot, Pfam,) containing gene function annotation information.

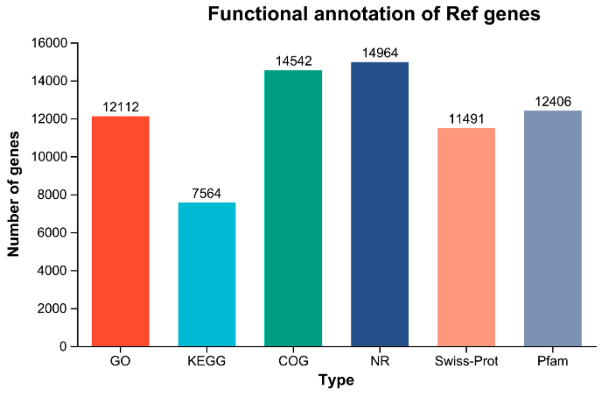

Figure S2. Determination of CYP and GST enzyme activities in midgut and fat body.

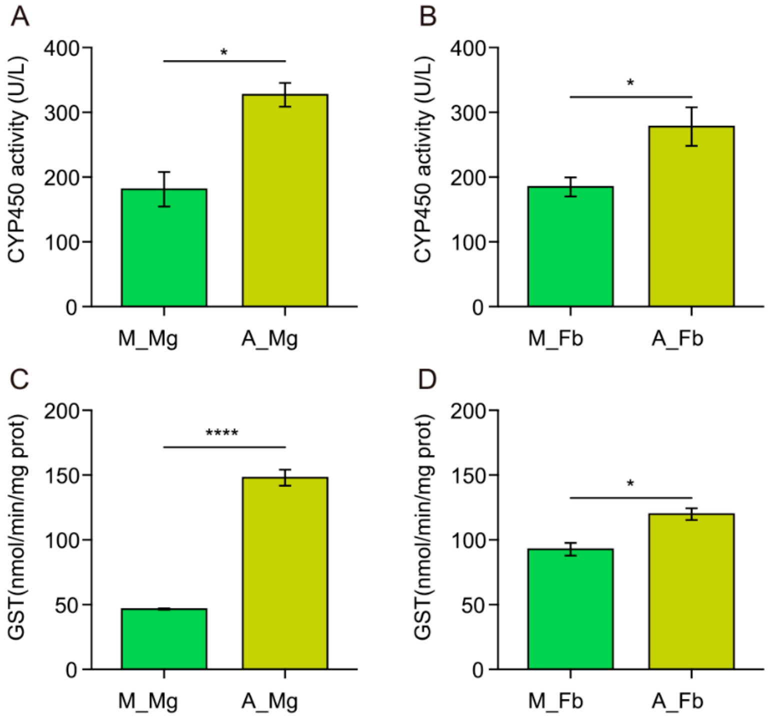

Midgut (Mg); fat body (Fb).

Figure S3. Heat map of hierarchical clustering analysis of differential metabolites. Each column represents one sample, and each single row represents one metabolite. Red to dark blue indicates abundance changes from high to low.

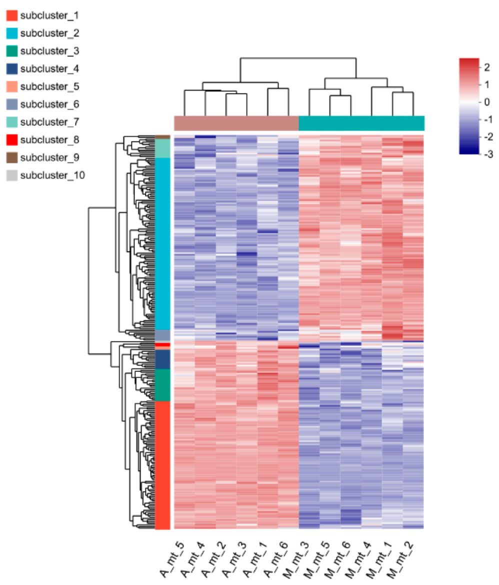

Table S1 Statistical data for transcriptome sample sequencing.

Table S2 List of differentially expressed genes (DEGs) in the upward curly section (c), upward straight section (up), and downward section (down) of the Malpighian tubules identified in the transcriptome analyses.

Table S3 List of differential metabolites identified in the metabolome analyses.

Table S4 Expression levels of the genes involved in the ribosome biogenesis. The gene expression level was calculated by TPM method. "+" represents up-regulation. "*" represents a differentially expressed gene (DEG). Log_2FC: log_2 (fold change).

[a] The value was calculated by log_2 (C-A-TPM/C-M-TPM)

[b] The value was calculated by log_2 (UP-A-TPM/UP-M-TPM)

[c] The value was calculated by log_2 (DOWN-A-TPM/DOWN-M-TPM)

Table S5 List of primers used in RT-qPCR.
